# Genomic characterization of ST38 NDM-5-producing *Escherichia coli* isolates from an outbreak in the Czech Republic

**DOI:** 10.1128/aac.00133-24

**Published:** 2024-04-16

**Authors:** Katerina Chudejova, Tsolaire Sourenian, Jana Palkovicova, Katarina Stredanska, Lucie Nechutna, Katerina Vlkova, Vendula Studentova, Working Group for Monitoring of Antibiotic Resistance, Marian Glasnak, Jaroslav Hrabak, Costas C. Papagiannitsis, Monika Dolejska, Ibrahim Bitar

**Affiliations:** 1 Rudolf's and Stefanie's Hospital in Benesov, Benesov, Czechia; 2 Laboratory Ifcor, Brno, Czechia; 3 University Hospital Brno, Brno, Czechia; 4 Laboratory Spadia, Brno, Czechia; 5 University Hospital sv. Anna, Brno, Czechia; 6 Laboratory Bio-Plus, Brno, Czechia; 7 Hospital Ceska Lipa, Ceska Lipa, Czechia; 8 Hospital Ceske Budejovice, Ceske Budejovice, Czechia; 9 Hospital Havlickuv Brod, Havlickuv Brod, Czechia; 10 University Hospital Hradec Kralove, Hradec Kralove, Czechia; 11 Laboratory Privatni mikrobiologicka laborator, Hradec Kralove, Czechia; 12 Laboratory Dia-Gon MP, Cheb, Czechia; 13 Laboratory Synlab, Chomutov, Czechia; 14 Hospital Chrudim, Chrudim, Czechia; 15 Hospital Jicin, Jicin, Czechia; 16 Hospital Jihlava, Jihlava, Czechia; 17 Hospital Jindrichuv Hradec, Jindrichuv Hradec, Czechia; 18 Hospital Karlovy Vary, Karlovy Vary, Czechia; 19 Hospital Kladno, Kladno, Czechia; 20 Hospital Klatovy, Klatovy, Czechia; 21 Hospital Kolin, Kolin, Czechia; 22 Hospital Kromeriz, Kromeriz, Czechia; 23 Hospital Kyjov, Kyjov, Czechia; 24 Hospital Liberec, Liberec, Czechia; 25 Hospital Litomysl, Litomysl, Czechia; 26 Hospital Mlada Boleslav, Mlada Boleslav, Czechia; 27 Hospital Most, Most, Czechia; 28 Hospital Nachod, Nachod, Czechia; 29 Hospital Nove Mesto na Morave, Nove Mesto na Morav, Czechia; 30 Laboratory Agel, Novy Jicin, Czechia; 31 University Hospital Olomouc, Olomouc, Czechia; 32 Hospital Opava, Opava, Czechia; 33 Laboratory Spadia, Ostrava, Czechia; 34 Laboratory Agel, Ostrava, Czechia; 35 Institute for Public Health, Ostrava, Czechia; 36 Hospital Pardubice, Pardubice, Czechia; 37 Hospital Pisek, Pisek, Czechia; 38 Hospital PRIVAMED, Plzen, Czechia; 39 University Hospital Pilsen, Plzen, Czechia; 40 General University Hospital, Prague, Czechia; 41 Hospital Na Homolce, Prague, Czechia; 42 IKEM, Prague, Czechia; 43 Laboratory Ceska laboratorni, Prague, Czechia; 44 Laboratory CityLab, Prague, Czechia; 45 Laboratory Spadia, Prague, Czechia; 46 Laboratory Synlab, Prague, Czechia; 47 Military University Hospital, Prague, Czechia; 48 "National Reference Laboratory for Antibiotics, National Institute of Public Health", Prague, Czechia; 49 Thomayer University Hospital, Prague, Czechia; 50 University Hospital Bulovka, Prague, Czechia; 51 University Hospital Kralovske Vinohrady, Prague, Czechia; 52 University Hospital Motol, Prague, Czechia; 53 Hospital Pribram, Pribram, Czechia; 54 AGEL Hospital Prostejov, Prostejov, Czechia; 55 Laboratory Agel, Sternberk, Czechia; 56 Hospital Strakonice, Strakonice, Czechia; 57 Hospital Sumperk, Sumperk, Czechia; 58 Hospital Tabor, Tabor, Czechia; 59 Hospital Trebic, Trebic, Czechia; 60 Laboratory Agel, Trinec, Czechia; 61 Hospital Trutnov, Trutnov, Czechia; 62 Hospital Uherske Hradiste, Uherske Hradiste, Czechia; 63 Laboratory Ifcor, Uherske Hradiste, Czechia; 64 Masaryk Hospital Usti nad Labem, Usti nad Labem, Czechia; 65 Usti nad Orlici Hospital, Usti nad Orlici, Czechia; 66 Hospital Vsetin, Vsetin, Czechia; 67 Hospital Vyskov, Vyskov, Czechia; 68 Bata's Hospital, Zlin, Czechia; 69 Hospital Znojmo, Znojmo, Czechia; 1 Department of Microbiology, Faculty of Medicine, University Hospital in Pilsen, Charles University, Pilsen, Czech Republic; 2 Central European Institute of Technology, University of Veterinary Sciences Brno, Brno, Czech Republic; 3 Department of Microbiology, University Hospital of Larissa, Larissa, Greece; 4 Department of Biology and Wildlife Diseases, Faculty of Veterinary Hygiene and Ecology, University of Veterinary Sciences Brno, Brno, Czech Republic; 5 Department of Clinical Microbiology and Immunology, Institute of Laboratory Medicine, The University Hospital Brno, Brno, Czech Republic; Shionogi, Inc., Florham Park, New Jersey, USA

**Keywords:** *Escherichia coli*, ST38, *bla*_NDM-5_

## Abstract

A 2-year national genomic screening in the Czech Republic identified a notable prevalence of the New Delhi metallo-β-lactamase 5 (NDM-5)-producing *Escherichia coli* sequence type 38 (ST38) in the city of Brno. Forty-two ST38 *E. coli* isolates harbored the *bla*_NDM-5_ gene on the chromosome. Virulence factors confirmed the persistence of these isolates through biofilm formation. Single Nucleotide Polymorphisms (SNPs)-based phylogeny and CRISPR assay typing showed minimal genomic variations, implying a clonally driven outbreak. Results suggest that this high-risk clone may impose a nationwide problem.

## INTRODUCTION

During an ongoing nationwide genomic surveillance of carbapenemase-producing Enterobacterales in the Czech Republic, a high number of NDM-5-producing *Escherichia coli* isolates belonging to the sequence type 38 (ST38) have been identified in Brno. Therefore, the study aims to investigate this outbreak and characterize these strains on a genomic level to understand the features that makes this clone persistent and virulent.

**Fig 1:**
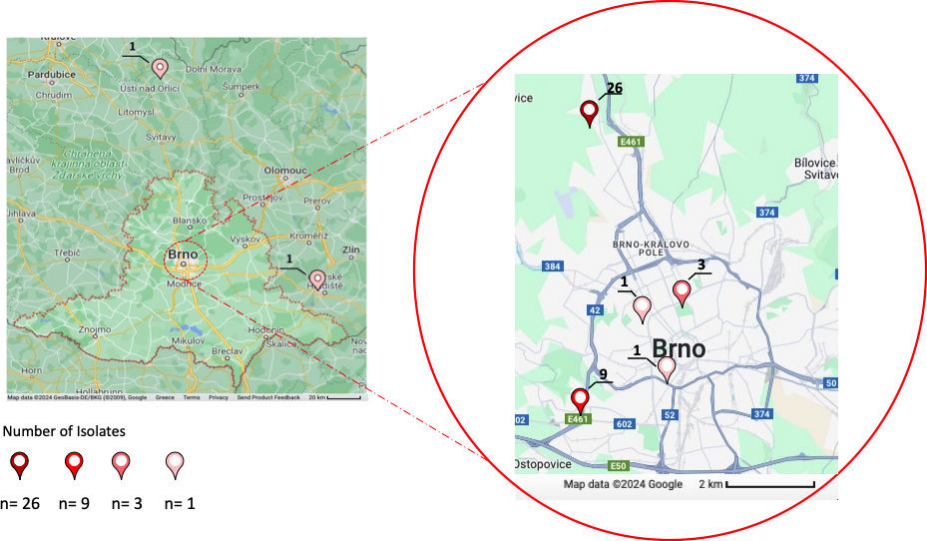
The 2D map of Brno and adjacent regions showing the number of detected isolates identified as NDM-5-producing ST38 *E. coli* strains. Dark red points correspond to 26 isolates, light red to 9, dark pink to 3, and light pink to 1.

Between 2020 and 2022, all NDM-5-producing *E. coli* ST38 isolates were detected (total number of 42) from seven hospitals/clinics in Brno (except for two isolates that came from adjacent areas) (Fig. 1; Table S1).

Antibiotic susceptibility testing was done for all isolates using broth microdilution assay, and results were interpreted according to the European Committee on Antimicrobial Susceptibility Testing (EUCAST) breakpoints 2024 (http://www.eucast.org/). All isolates showed high antibiotic resistance against most antibiotics including carbapenems, yet remained susceptible against tigecycline, colistin (except two isolates), and tetracycline in some isolates. Variations in MICs against meropenem were investigated further. Premature stop codons (in amino acid positions 13, 44, and 284) were detected in the outer membrane porin F (OmpF) in isolates with higher meropenem MICs (Table S2), while no differences were found in the deduced protein sequences of OmpA and OmpC among our isolates. Based on the geographic distribution (hospitals and wards) and antibiotic susceptibility profiles, a total of 26 isolates were selected as representatives and were eventually subjected to whole genome sequencing (WGS) using NovaSeq 6000 (Illumina, San Diego, CA, USA). Out of these, eight isolates were further characterized using long-read sequencing with Sequel I (Pacific Biosciences, Menlo Park, CA, USA), and data were analyzed as described previously (1). Long-read sequencing analysis showed that the *bla*_NDM-5_ gene was located on the chromosome in all the eight sequenced isolates. Furthermore, *in silico* scaffolding using Bandage (2) was performed on the short-read assemblies, which showed the *bla*_NDM-5_ gene was also located on the chromosome in the remaining 18 isolates (Table S3). All NDM-5-producing *E. coli* ST38 isolates were detected in the same region of the Czech Republic (Brno city and its surroundings), and the presence of the *bla*_NDM-5_ gene on the chromosome suggests a clonal outbreak.

**Fig 2:**
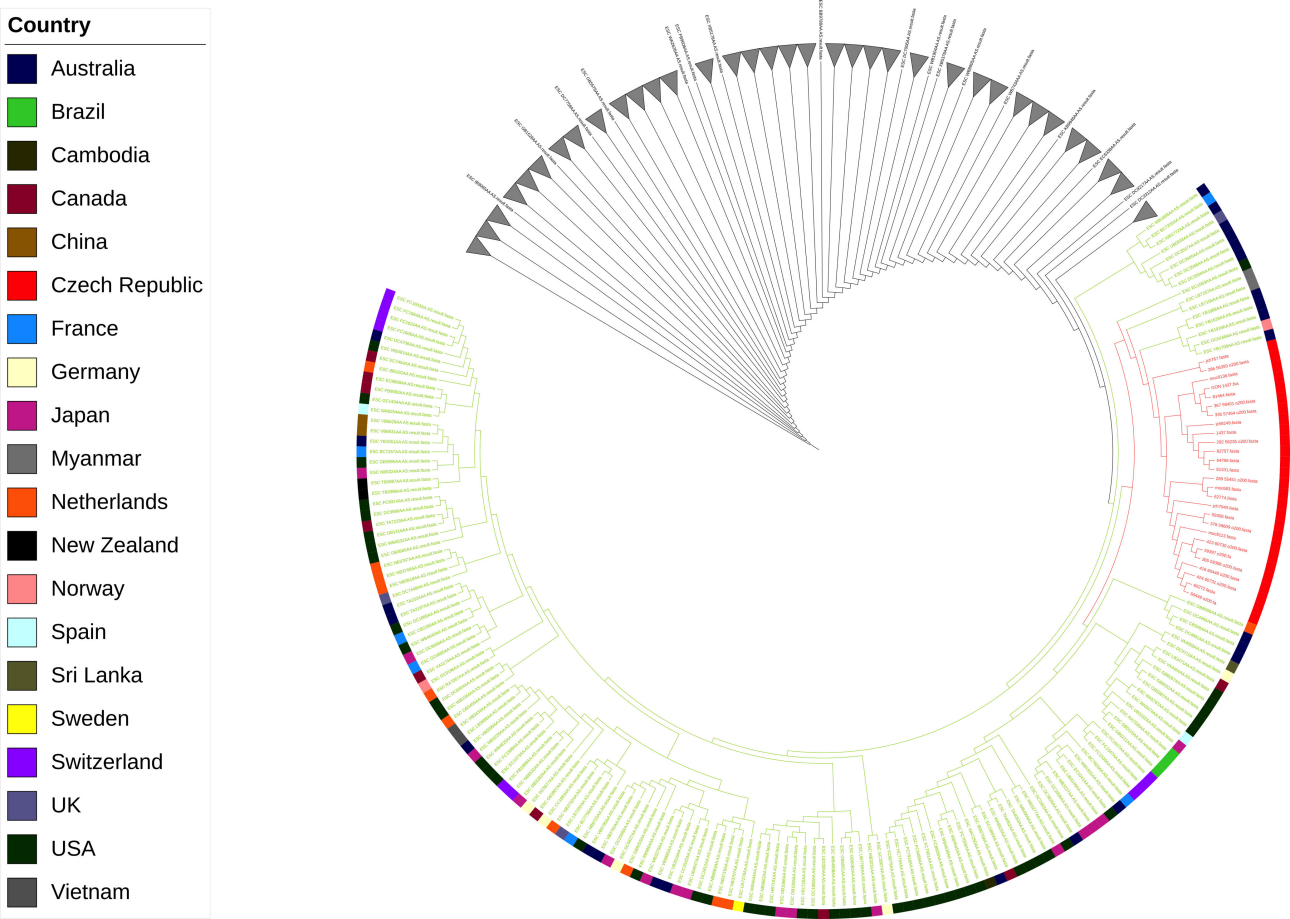
The phylogenetic analysis of the clade harboring Czech isolates. The Czech isolates node is colored in red. The outer membrane ring corresponds to the isolate’s reporting country.

The genomic relatedness of the Czech isolates was evaluated against all the ST38 *E. coli* genomes (*n* = 2,322) in the Enterobase database (https://enterobase.warwick.ac.uk/). Using Parsnp v1.2 from the Harvest suite (3), SNPs-based phylogeny was constructed on the 2,322 genomes along with the 26 genomes from this study using jch8249 as a reference. The unrooted phylogeny showed that all the Czech genomes (in this study) were grouped in one clade. All other clades were collapsed to have a more detailed view. The Czech genomes of this study clustered together in a subclade along with seven isolates from Australia (*n* = 4), Vietnam (*n* = 2), and Norway (*n* = 1) (Fig. 2). Interestingly, only the Czech isolates and the seven isolates mentioned above had the *bla*_NDM-5_ gene.

Furthermore, the clonality of the isolates in this outbreak was assessed (and the seven genomes detected in the same subclade) using Snippy v4.5 (4) as described before (5), using the genome of jch8249 as a reference (selected randomly). The results showed that the number of SNPs in both coding and non-coding regions varied from 2 to 16 SNPs between the isolates in this study and from 15 to 25 SNPs in the seven genomes found in the same subclade (Table S4), confirming the clonality of the isolates causing the outbreak in general and more specifically in Brno. The genomes from Vietnam had the least SNPs compared to the reference (15) followed by the genome from Norway (16). Remarkably, in some cases, it detected the presence of the same isolate in multiple patients. In SurGal Clinic Brno, the two strains (56235 and 57464) were isolated 1 month apart yet had the same two SNPs when compared against the reference. Also, 55393 and jch767, isolated from Syn Lab and University Hospital Brno, respectively, shared the same five SNPs and were isolated 6 months apart. Finally, 60272 and 604647, which were isolated from University Hospital Brno and Chronicare Mund Brno, respectively, on the same day, shared the same nine SNPs. These results showed the high clonality of the strains circulating within that region.

Moreover, the clonality of the strains was assessed by assessing the CRISPR array sequences. The spacers in CRISPR arrays were added in chronological order; therefore, it could be used to trace the clonality and the origin of the isolates and define them as a population of strains that were subjected to the same environmental conditions including geographic location (region) and community/hospital settings (6). The genome assemblies of this study along with the seven genomes in the same subclade were uploaded to CRISPRCasFinder (https://crisprcas.i2bc.paris-saclay.fr/) which showed that all the strains had the CRISPR/Cas I-E type with two CRISPR arrays (7). The CRISPR arrays of all isolates were identical (100%). This implies the presence of a possible single case, which was the ancestor of this outbreak.

In all the Czech isolates, an ≈27-kb region carrying *bla*_NDM-5_ was found. This region also harbored other antibiotic resistance genes including *aadA2*, *mphA*, *sul1*, *dfrA12*, and *ermB* for resistance to aminoglycosides, macrolides, sulphonamides, and trimethoprim. BLASTn comparison showed that the region was identical in all the studied isolates and the seven related genomes mentioned earlier and exhibited high similarity with sequences from plasmids, like a 161,700-bp IncF (F90:A2:B33) plasmid (pNDM_p30_L1, accession number CP085061.1) isolated from human *E. coli* in the UK in 2021 and a 148,746-bp IncF (F90:A2:B20) plasmid (pIsolateD_A, accession number CP135489.1) isolated from human *E. coli* in Canada in 2023. The ≈27 kb region was flanked by two IS*26* in the same orientation. No direct repeats were found in the insertion site suggesting a possible homologous recombination. The region was blasted on PHASTER (https://phaster.ca/) (8) but no phages were found. Long sequencing reads revealed that the insertion site of this region is in the same position in the chromosomes. Thus, the plasmid region seemed to be successfully integrated into the chromosome of NDM-5-producing *E. coli* ST38 via homologs recombination or transposition and remained stable as well since the clone itself appeared to be successful.

Furthermore, the WGS data were analyzed with the virulence factor database (VFDB) (http://www.mgc.ac.cn/VFs/) (9). The 26 isolates from this study carried similar virulence genes (with some slight variations). The observed virulence genes were associated with adherence and fimbrial adherence determinants, autotransporters, iron uptake, invasion, T6SS secretion system, and hemolysin A toxin. Phenotypically, biofilm formation was assessed, as described previously (10). Results showed that 13 out of 26 (50%) isolates produced strong biofilm, 9 (35%) were moderate, and 4 (15%) were considered weak biofilm producers. These virulence profiles seem to be highly conserved among the ST38 genomes. Biofilm formation was achieved after 3 days of incubation and significantly reached maximum adherence after 6 days of incubation. The one-way analysis of variance test showed a significant association (*P* value <0.05) between the incubation time and the biofilm formation (Fig. S1).

In conclusion, *E. coli* ST38 is a high-risk clone reported in many countries to carry different antibiotic resistance determinants (11–13). Here, we showed the acquisition of NDM-5 carbapenemase by this clone and its successful spread in one region of the Czech Republic. Recently, similar reports from different countries, especially Germany, have been published (11), indicating the dissemination of NDM-5 carbapenemase in various *E. coli* clonal lineages. Our results showed that this outbreak was clonally driven with minimal genomic variation between the isolates. The *bla*_NDM-5_ was chromosomally encoded, and the genetic environment around it was likely acquired from previously identified plasmids. The region seemed to be stable and harbored multiple insertion sequences and antibiotic resistance genes which implies the possibility of acquiring more resistance genes and integrating to other replicons and/or clones. Additionally, we showed that the presence of premature stop codons in the sequence of *ompF* contributed to the increased meropenem MICs. The isolates show high antibiotic resistance and virulence profiles confirming the successful dissemination and persistence in Brno for over a year. Further monitoring of this clone is needed since the situation infers a possible outbreak on a national scale.

## Data Availability

The nucleotide sequences of the isolates have been deposited in GenBank under the BioProject number PRJNA980112.

## SUPPLEMENTAL MATERIAL

Figure S1 aac.00133-24-s0001.pdf Figure S1: Biofilm assay; values of absorbance at 570 nm obtained for E. coli ST38 isolates after 1, 3 and 6 days of incubation.

Table S1 aac.00133-24-s0002.docx The descriptive data of the ST38 NDM-5-producing E. coli isolates from the Czech Republic. This data includes isolation date, specimen type, hospital, and ward of the isolation.

Table S2 aac.00133-24-s0003.docx Antibiotic susceptibility profiles and premature stop codon in outer membrane protein for the ST38 NDM-5 producing E. coli isolates from the Czech Republic.

Table S3 aac.00133-24-s0004.docx WGS analysis including plasmid replicons and Resistance genes in all the sequenced Czech isolates.

Table S4 aac.00133-24-s0005.xlsx Snippy analysis showing the SNPs detected in the genomes compared to the reference genome jch8249. Amino acid substitutions and their effects on the product are also shown.

ASM does not own the copyrights to Supplemental Material that may be linked to, or accessed through, an article. The authors have granted ASM a non-exclusive, world-wide license to publish the Supplemental Material files. Please contact the corresponding author directly for reuse.
