# Supplementary material for: Genomic characterization of ST38 NDM-5-producing Escherichia coli isolates from an outbreak in the Czech Republic
Source: Antimicrob Agents Chemother. 2024 Apr 16;68(6):e00133-24. doi: 10.1128/aac.00133-24 (PMC11620504; doi:10.1128/aac.00133-24)
Supplement: Figure S1 — Biofilm assay; values of absorbance at 570 nm obtained for E. coli ST38 isolates after 1, 3 and 6 days of incubation. [file aac.00133-24-s0001.pdf]

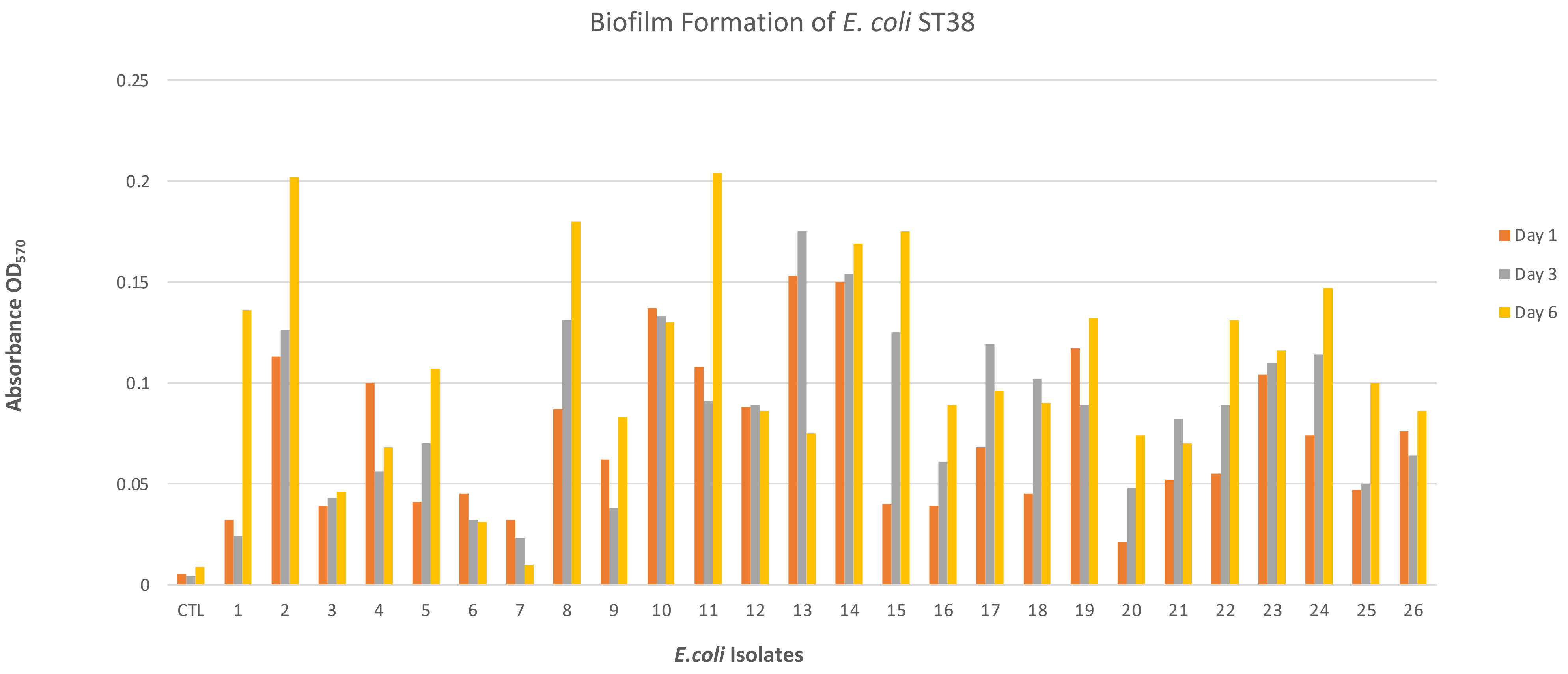

Figure S1: Biofilm assay; values of absorbance at 570 nm obtained for *E. coli* ST38 isolates after 1, 3 and 6 days of incubation.
