# Supplementary material for: Genomic characterization of ST38 NDM-5-producing Escherichia coli isolates from an outbreak in the Czech Republic
Source: Antimicrob Agents Chemother. 2024 Apr 16;68(6):e00133-24. doi: 10.1128/aac.00133-24 (PMC11620504; doi:10.1128/aac.00133-24)
Supplement: Table S1 — The descriptive data of the ST38 NDM-5-producing E. coli isolates from the Czech Republic. This data includes isolation date, specimen type, hospital, and ward of the isolation. [file aac.00133-24-s0002.docx]

**Table S1:** The descriptive data of the ST38 NDM-5-producing *E. coli* isolates from the Czech Republic. This data includes isolation date, specimen type, hospital, and ward of the isolation.

| **Sample ID** | **Isolation date** | **Specimen** | **Ward** | **Hospital** |
| --- | --- | --- | --- | --- |
| 55393 | 07.01.2020 | wound swab | General ambulance | Synlab, Brno |
| 55451 | 09.01.2020 | urine | Trauma surgery | University Hospital Brno |
| 56235 | 11.02.2020 | sputum | Department of Anaesthesiology and Resuscitation | SurGal clinic Brno |
| 57464 | 12.03.2020 | urine | Department of Anaesthesiology and Resuscitation | SurGal clinic Brno |
| 59306 | 19.08.2020 | urine - permanent urinary catheter | Department of Anaesthesiology and Resuscitation | University Hospital St. Anne |
| 59396 | 03.08.2020 | sputum | Follow-up intensive care | Chronicare Mund Brno |
| 59397 | 03.08.2020 | central venous catheter | Follow-up intensive care | Chronicare Mund Brno |
| 59401 | 11.08.2020 | urine | Department of Anaesthesiology and Resuscitation | SurGal Clinic Brno |
| 59609 | 01.09.2020 | urine | Follow-up intensive care | Chronicare Mund Brno |
| 60272 | 05.10.2020 | oral cavity swab | Trauma surgery | University Hospital Brno |
| 60448 | 05.10.2020 | urine | Follow-up intensive care | Chronicare Mund Brno |
| 60449 | 18.10.2020 | sputum | Follow-up intensive care | Chronicare Mund Brno |
| 60730 | 02.11.2020 | urine | Follow-up intensive care | Chronicare Mund Brno |
| 60731 | 24.10.2020 | urine | Follow-up intensive care | Chronicare Mund Brno |
| 61331 | 02.12.2020 | urine | Follow-up intensive care | Chronicare Mund Brno |
| 61464 | 10.12.2020 | urine | General clinic | Hospital Pardubickeho kraje a.s., Žamberk |
| 62757 | 02.03.2021 | bronchoalveolar lavage | Pulmonary clinic | Hospital Uherske Hradiste |
| 62774 | 02.03.2021 | decubitus | Follow-up intensive care | Chronicare Mund Brno |
| 64796 | 03.05.2021 | urine | Follow-up intensive care | Chronicare Mund Brno |
| GON1437 | 28.06.2021 | urethra swab | Clinic of lung diseases and tuberculosis | University Hospital Brno |
| JCH7040 | 22.06.2021 | perianal swab | Neurosurgery clinic | University Hospital Brno |
| JCH767 | 20.01.2020 | wound swab | Clinic of burns and plastic surgery | University Hospital Brno |
| JCH8249 | 24.07.2021 | perianal swab | Department of Anaesthesiology and Resuscitation | University Hospital Brno |
| MOC653 | 09.01.2020 | urine | Trauma surgery | University Hospital Brno |
| MOC8113 | 14.04.2022 | urine | Clinic of lung diseases and tuberculosis | University Hospital Brno |
| MOC9136 | 28.04.2022 | urine | Trauma surgery | University Hospital Brno |
| 60 071 | 24.09.2020 | urine | Follow-up intensive care | Chronicare Mund Brno |
| 60 072 | 25.09.2020 | urine | Follow-up intensive care | Chronicare Mund Brno |
| 61 334 | 07.12.2020 | sputum | Follow-up intensive care | Chronicare Mund Brno |
| 61 862 | 04.01.2021 | sputum | Follow-up intensive care | Chronicare Mund Brno |
| 61 860 | 04.01.2021 | urine | Follow-up intensive care | Chronicare Mund Brno |
| 61 861 | 04.01.2021 | urine | Follow-up intensive care | Chronicare Mund Brno |
| 62 508 | 16.02.2021 | urine | Follow-up intensive care | Chronicare Mund Brno |
| 63 133 | 01.03.2021 | urine | Follow-up intensive care | Chronicare Mund Brno |
| 63 132 | 01.03.2021 | urine | Follow-up intensive care | Chronicare Mund Brno |
| 62 758 | 02.03.2021 | urine | Follow-up intensive care | Chronicare Mund Brno |
| 63 134 | 11.03.2021 | decubitus | Follow-up intensive care | Chronicare Mund Brno |
| 63 599 | 26.03.2021 | urine | Follow-up intensive care | Chronicare Mund Brno |
| 63 600 | 29.03.2021 | urine | Follow-up intensive care | Chronicare Mund Brno |
| 63 601 | 29.03.2021 | urine | Follow-up intensive care | Chronicare Mund Brno |
| 63 967 | 03.04.2021 | urine | Follow-up intensive care | Chronicare Mund Brno |
| 64 478 | 13.04.2021 | urine | Follow-up intensive care | Chronicare Mund Brno |
