# Supplementary material for: Genomic characterization of ST38 NDM-5-producing Escherichia coli isolates from an outbreak in the Czech Republic
Source: Antimicrob Agents Chemother. 2024 Apr 16;68(6):e00133-24. doi: 10.1128/aac.00133-24 (PMC11620504; doi:10.1128/aac.00133-24)
Supplement: Table S2 — Antibiotic susceptibility profiles and premature stop codon in outer membrane protein for the ST38 NDM-5 producing E. coli isolates from the Czech Republic. [file aac.00133-24-s0003.docx]

Table S2: Antibiotic susceptibility profiles and premature stop codon in outer membrane protein for the ST38 NDM-5 producing *E. coli* isolates from the Czech Republic.

|  | **MIC (mg/L)** | | | | | | | | | | | | | | | | | | | | | | | |  |
| --- | --- | --- | --- | --- | --- | --- | --- | --- | --- | --- | --- | --- | --- | --- | --- | --- | --- | --- | --- | --- | --- | --- | --- | --- | --- |
| **Isolate** | **Amp** | **Sam** | **Cfz** | **Cxm** | **Atm** | **Gen** | **Amk** | **Cst** | **Sxt** | **Cip** | **Chl** | **Tet** | **Pip** | **Tzp** | **Ctx** | **Caz** | **Cfp** | **Cps** | **Fep** | **Mem** | **Etp** | **Tgc** | **Net** | **Tob** | ***ompF**** |
| **55393** | >128 | >128 | >16 | >64 | 2 | >32 | >64 | <0,125 | >4 | >8 | 8 | 2 | 64 | 32 | >8 | >16 | 64 | 32 | 4 | 1 | 2 | 0,25 | >16 | >8 | NA |
| **55451** | >128 | >128 | >16 | >64 | 1 | >32 | >64 | <0,125 | >4 | >8 | 8 | 4 | 64 | 64 | >8 | >16 | 64 | 32 | 4 | 2 | >2 | 0,25 | >16 | >8 | NA |
| **56235** | >128 | >128 | >16 | >64 | 2 | >32 | >64 | <0,125 | >4 | >8 | 16 | 2 | 128 | 64 | >8 | >16 | >64 | 64 | 8 | 2 | 2 | 0,25 | >16 | >8 | NA |
| **57464** | >128 | >128 | >16 | >64 | 1 | >32 | >64 | <0,125 | >4 | >8 | 16 | 2 | 128 | 128 | >8 | >16 | >64 | 64 | 8 | 1 | 2 | 0,125 | >16 | >8 | NA |
| **59306** | >128 | >128 | >16 | >64 | 16 | >32 | >64 | <0,125 | >4 | >8 | >32 | >32 | >128 | >128 | >8 | >16 | >64 | >64 | >16 | 16 | >2 | 0,125 | >16 | >8 | *13 |
| **59396** | >128 | >128 | >16 | >64 | 2 | >32 | >64 | <0,125 | >4 | >8 | 16 | 4 | 128 | 128 | >8 | >16 | >64 | >64 | 16 | 4 | >2 | 0,125 | >16 | >8 | NA |
| **59397** | >128 | >128 | >16 | >64 | 2 | >32 | >64 | <0,125 | >4 | >8 | 16 | 4 | 64 | 32 | >8 | >16 | 64 | >64 | 8 | 4 | >2 | 0,25 | >16 | >8 | *13 |
| **59401** | >128 | 128 | >16 | >64 | 0,5 | >32 | >64 | <0,125 | >4 | >8 | 8 | 1 | 128 | 64 | >8 | >16 | 64 | 16 | 4 | 0,5 | 0,5 | 0,25 | >16 | >8 | NA |
| **59609** | >128 | >128 | >16 | >64 | 2 | >32 | >64 | <0,125 | >4 | >8 | 16 | 2 | 128 | 128 | >8 | >16 | >64 | >64 | 16 | 8 | >2 | 0,25 | >16 | >8 | *13 |
| **60272** | >128 | >128 | >16 | >64 | 16 | >32 | >64 | <0,125 | >4 | >8 | >32 | >32 | 128 | 128 | >8 | >16 | >64 | >64 | 8 | 4 | >2 | 0,25 | >16 | >8 | *13 |
| **60448** | >128 | >128 | >16 | >64 | >16 | >32 | >64 | <0,125 | >4 | >8 | >32 | >32 | >128 | >128 | >8 | >16 | >64 | >64 | >16 | 8 | >2 | 0,125 | >16 | >8 | *13 |
| **60449** | >128 | >128 | >16 | >64 | 16 | >32 | >64 | 0,25 | >4 | >8 | >32 | >32 | >128 | 128 | >8 | >16 | >64 | 64 | 16 | 4 | >2 | 0,25 | >16 | >8 | *13 |
| **60730** | >128 | >128 | >16 | >64 | 16 | >32 | >64 | <0,125 | >4 | >8 | >32 | >32 | >128 | 128 | >8 | >16 | >64 | >64 | 16 | 4 | >2 | 0,125 | >16 | >8 | *13 |
| **60731** | >128 | >128 | >16 | >64 | >16 | >32 | >64 | <0,125 | >4 | >8 | >32 | >32 | >128 | >128 | >8 | >16 | >64 | >64 | 16 | 4 | >2 | 0,25 | >16 | >8 | *13 |
| **61331** | >128 | >128 | >16 | >64 | 1 | >32 | >64 | <0,125 | >4 | >8 | 8 | 2 | 64 | 64 | >8 | >16 | >64 | >64 | 8 | 2 | 2 | 0,125 | >16 | >8 | NA |
| **61464** | >128 | >128 | >16 | >64 | 2 | >32 | >64 | <0,125 | >4 | >8 | 4 | 1 | 128 | 128 | >8 | >16 | >64 | >64 | 4 | 4 | >2 | 0,25 | >16 | >8 | NA |
| **62757** | >128 | >128 | >16 | >64 | 1 | >32 | >64 | <0,125 | >4 | >8 | 16 | 2 | 128 | 64 | >8 | >16 | >64 | >64 | 8 | 4 | >2 | 0,125 | >16 | >8 | *284 |
| **62774** | >128 | >128 | >16 | >64 | 8 | >32 | >64 | 0,25 | >4 | >8 | >32 | >32 | >128 | 128 | >8 | >16 | >64 | 64 | 8 | 0,5 | 2 | 0,125 | >16 | >8 | NA |
| **64796** | >128 | >128 | >16 | >64 | 8 | >32 | >64 | <0,125 | >4 | >8 | 8 | 4 | >128 | >128 | >8 | >16 | >64 | >64 | 16 | 2 | >2 | 0,25 | >16 | >8 | NA |
| **gon1437** | >128 | >128 | >16 | >64 | 1 | 0,5 | 2 | <0,125 | >4 | >8 | 4 | 2 | 128 | 64 | >8 | >16 | >64 | 64 | 8 | 2 | 2 | 0,25 | 0,5 | 0,5 | NA |
| **jch7040** | >128 | >128 | >16 | >64 | 16 | >32 | >64 | <0,125 | >4 | >8 | >32 | >32 | >128 | >128 | >8 | >16 | >64 | >64 | >16 | 16 | >2 | 0,25 | >16 | >8 | *13 |
| **jch767** | >128 | >128 | >16 | >64 | 1 | >32 | >64 | <0,125 | >4 | >8 | 8 | 2 | 64 | 64 | >8 | >16 | >64 | 64 | 8 | 1 | 2 | 0,25 | >16 | >8 | NA |
| **jch8249** | >128 | >128 | >16 | >64 | 4 | >32 | >64 | <0,125 | >4 | >8 | 16 | 4 | 128 | 64 | >8 | >16 | >64 | >64 | 16 | 8 | >2 | 0,25 | >16 | >8 | *44 |
| **moc653** | >128 | >128 | >16 | >64 | 1 | >32 | >64 | <0,125 | >4 | >8 | 8 | 4 | 128 | 64 | >8 | >16 | >64 | >64 | 8 | 2 | 1 | 0,25 | >16 | >8 | NA |
| **moc8113** | >128 | >128 | >16 | >64 | 16 | >32 | >64 | 4 | >4 | >8 | >32 | >32 | >128 | 128 | >8 | >16 | >64 | >64 | 16 | 8 | >2 | 0,125 | >16 | >8 | *13 |
| **moc9136** | >128 | >128 | >16 | >64 | 4 | >32 | >64 | <0,125 | >4 | >8 | 8 | 4 | >128 | 64 | >8 | >16 | >64 | >64 | 16 | 4 | >2 | 0,125 | >16 | >8 | *44 |
| **60071** | >128 | >128 | >16 | >64 | 16 | >32 | >64 | <0,125 | >4 | >8 | >32 | >32 | 128 | 128 | >8 | >16 | >64 | >64 | >16 | 8 | >2 | 0,25 | >16 | >8 | NS |
| **60072** | >128 | >128 | >16 | >64 | 1 | >32 | >64 | <0,125 | >4 | >8 | 16 | 4 | 128 | 64 | >8 | >16 | >64 | >64 | 8 | 8 | >2 | 0,25 | >16 | >8 | NS |
| **61334** | >128 | >128 | >16 | >64 | 1 | >32 | >64 | <0,125 | >4 | >8 | 8 | 4 | >128 | 64 | >8 | >16 | >64 | 64 | 16 | 2 | 2 | 0,25 | >16 | >8 | NS |
| **61862** | >128 | >128 | >16 | >64 | 2 | >32 | >64 | <0,125 | >4 | >8 | 16 | 4 | 64 | 64 | >8 | >16 | 64 | 64 | 4 | 4 | >2 | 0,5 | >16 | >8 | NS |
| **61860** | >128 | >128 | >16 | >64 | 8 | >32 | >64 | <0,125 | >4 | >8 | >32 | 32 | >128 | 128 | >8 | >16 | >64 | 32 | 8 | 1 | 1 | 0,25 | >16 | >8 | NS |
| **61861** | >128 | >128 | >16 | >64 | 2 | >32 | >64 | 4 | >4 | >8 | 8 | 2 | >128 | 64 | >8 | >16 | >64 | >64 | 16 | 2 | >2 | 0,125 | >16 | >8 | NS |
| **62508** | >128 | >128 | >16 | >64 | 2 | >32 | >64 | <0,125 | >4 | >8 | 16 | 2 | 64 | 64 | >8 | >16 | >64 | >64 | 16 | 4 | >2 | 0,125 | >16 | >8 | NS |
| **63133** | >128 | >128 | >16 | >64 | 2 | >32 | >64 | <0,125 | >4 | >8 | 32 | 4 | 64 | 128 | >8 | >16 | >64 | >64 | 16 | 8 | >2 | 0,125 | >16 | >8 | NS |
| **63132** | >128 | >128 | >16 | >64 | 16 | >32 | >64 | 0,25 | >4 | >8 | >32 | >32 | >128 | 128 | >8 | >16 | >64 | >64 | 16 | 8 | >2 | 0,125 | >16 | >8 | NS |
| **62758** | >128 | >128 | >16 | >64 | 16 | >32 | >64 | <0,125 | >4 | >8 | >32 | >32 | >128 | >128 | >8 | >16 | >64 | >64 | >16 | 16 | >2 | 0,125 | >16 | >8 | NS |
| **63134** | >128 | >128 | >16 | >64 | 4 | >32 | >64 | <0,125 | >4 | >8 | 16 | 4 | 128 | 64 | >8 | >16 | >64 | >64 | 16 | 4 | >2 | 0,125 | >16 | >8 | NS |
| **63599** | >128 | >128 | >16 | >64 | 16 | >32 | >64 | <0,125 | >4 | >8 | >32 | >32 | >128 | >128 | >8 | >16 | >64 | >64 | >16 | 8 | >2 | 0,125 | >16 | >8 | NS |
| **63600** | >128 | >128 | >16 | >64 | 4 | >32 | >64 | <0,125 | >4 | >8 | 16 | 4 | 128 | 128 | >8 | >16 | >64 | >64 | 16 | 2 | >2 | 0,5 | >16 | >8 | NS |
| **63601** | >128 | >128 | >16 | >64 | 2 | >32 | >64 | <0,125 | >4 | >8 | 16 | 4 | >128 | 64 | >8 | >16 | >64 | >64 | 8 | 2 | >2 | 0,25 | >16 | >8 | NS |
| **63967** | >128 | >128 | >16 | >64 | 1 | >32 | >64 | <0,125 | >4 | >8 | 16 | 2 | 64 | 64 | >8 | >16 | >64 | 64 | 8 | >16 | >2 | 0,25 | >16 | >8 | NS |
| **64478** | >128 | >128 | >16 | >64 | >16 | >32 | >64 | <0,125 | >4 | >8 | >32 | >32 | >128 | 128 | >8 | >16 | >64 | >64 | >16 | 8 | >2 | 0,25 | >16 | >8 | NS |

Amp, ampicilin; Sam, ampicillin-sulbactam; Cfz, cefazolin; Cxm, cefuroxime; Atm, aztreonam; Gen, gentamicin; Amk, amikacin; Cst, colistin; Sxt, trimethoprim-sulfamethoxazole; Cip, ciprofloxacin; Chl, chloramphenicol; Tet, tetracycline; Pip, piperacillin; Tzp, piperacillin-tazobactam; Ctx, cefotaxime; Caz, ceftazidime; Cfp, cefoperazone; Cps, cefoperazone-sulbactam; Fep, cefepime; Mem, meropenem; Etp, ertapenem; Tgc, tigecycline; Net, netilmicin; Tob, tobramycin

ompF*; indicates the stop codon in the deduced protein sequence, NA; not applicable, NS; not sequenced.
