# Supplementary material for: Genomic characterization of ST38 NDM-5-producing Escherichia coli isolates from an outbreak in the Czech Republic
Source: Antimicrob Agents Chemother. 2024 Apr 16;68(6):e00133-24. doi: 10.1128/aac.00133-24 (PMC11620504; doi:10.1128/aac.00133-24)
Supplement: Table S3 — WGS analysis including plasmid replicons and Resistance genes in all the sequenced Czech isolates. [file aac.00133-24-s0004.docx]

Table S3: WGS analysis including plasmid replicons and Resistance genes in all the sequenced Czech isolates.

| **Sample ID** | **Replicons** | **Resistance genes** |
| --- | --- | --- |
| **55393** |  | *aadA2, aph(3'')-Ib, aph(6)-Id*, *bla*_CTX-M-14_, *bla*_NDM-5_, *bla*_TEM-1B_, *dfrA12, erm(B), mdf(A), mph(A), rmtB, sul1, sul2* |
| **55451** |  | *aadA2, aph(3'')-Ib, aph(6)-Id*, *bla*_CTX-M-14_, *bla*_NDM-5_, *bla*_TEM-1B_, *dfrA12, erm(B), mdf(A), mph(A), rmtB, sul1, sul2* |
| **56235** |  | *aadA2, aph(3'')-Ib, aph(6)-Id*, *bla*_CTX-M-14_, *bla*_NDM-5_, *bla*_TEM-1B_, *dfrA12, erm(B), mdf(A), mph(A), rmtB, sul1, sul2* |
| **57464** |  | *aadA2, aph(3'')-Ib, aph(6)-Id*, *bla*_CTX-M-14_, *bla*_NDM-5_, *bla*_TEM-1B_, *dfrA12, erm(B), mdf(A), mph(A), rmtB, sul1, sul2* |
| **59306** | IncHI2A, IncHI2 | *aac(6')-Ib-cr, aph(3'')-Ib, aph(6)-Id,* *bla*_CTX-M-14_, *bla*_NDM-5_, *bla*_OXA-1_, *bla*_TEM-1B_, catA1, *dfrA12, dfrA14, erm(B), mdf(A), mph(A), qnrB1, rmtB, sul1, sul2* |
| **59396** |  | *aph(3'')-Ib, aph(6)-Id*, *bla*_CTX-M-14_, *bla*_NDM-5_, *bla*_TEM-1B_, *dfrA12, erm(B), mdf(A), mph(A), rmtB, sul1, sul2* |
| **59397** |  | *aph(3'')-Ib, aph(6)-Id*, *bla*_CTX-M-14_, *bla*_NDM-5_, *bla*_TEM-1B_, *dfrA12, erm(B), mdf(A), mph(A), rmtB, sul1, sul2* |
| **59401** |  | *aadA2, aph(3'')-Ib, aph(6)-Id*, *bla*_CTX-M-14_, *bla*_NDM-5_, *bla*_TEM-1B_, *dfrA12, erm(B), mdf(A), mph(A), rmtB, sul1, sul2* |
| **59609** |  | *aph(3'')-Ib, aph(6)-Id*, *bla*_CTX-M-14_, *bla*_NDM-5_, *bla*_TEM-1B_, *dfrA12, erm(B), mdf(A), mph(A), rmtB, sul1, sul2* |
| **60272** | IncHI2A, IncHI2 | *aac(6')-Ib-cr, aph(3'')-Ib, aph(6)-Id,* *bla*_CTX-M-14_, *bla*_NDM-5_, *bla*_OXA-1_, *bla*_TEM-1B_, catA1, *dfrA12, dfrA14, erm(B), mdf(A), mph(A), qnrB1, rmtB, sul1, sul2* |
| **60448** | IncHI2A, IncHI2 | *aac(6')-Ib-cr, aph(3'')-Ib, aph(6)-Id*, *bla*_CTX-M-14_, *bla*_NDM-5_, *bla*_OXA-1_, *bla*_SHV-1_, *bla*_TEM-1B_, *catA1, dfrA12, dfrA14, erm(B), mdf(A), mph(A), qnrB1, rmtB, sul1, sul2* |
| **60449** | IncHI2A, IncHI2 | *aac(6')-Ib-cr, aph(3'')-Ib, aph(6)-Id,* *bla*_CTX-M-14_, *bla*_NDM-5_, *bla*_OXA-1_, *bla*_TEM-1B_, catA1, *dfrA12, dfrA14, erm(B), mdf(A), mph(A), qnrB1, rmtB, sul1, sul2* |
| **60730** | IncHI2A, IncHI2 | aac *aac(6')-Ib-cr, aph(3'')-Ib, aph(6)-Id,* *bla*_CTX-M-14_, *bla*_NDM-5_, *bla*_OXA-1_, *bla*_TEM-1B_, catA1, *dfrA12, dfrA14, erm(B), mdf(A), mph(A), qnrB1, rmtB, sul1, sul2* |
| **60731** | IncHI2A, IncHI2 | *aac(6')-Ib-cr, aph(3'')-Ib, aph(6)-Id,* *bla*_CTX-M-14_, *bla*_NDM-5_, *bla*_OXA-1_, *bla*_TEM-1B_, catA1, *dfrA12, dfrA14, erm(B), mdf(A), mph(A), qnrB1, rmtB, sul1, sul2* |
| **61331** |  | *aadA2, aph(3'')-Ib, aph(6)-Id*, *bla*_CTX-M-14_, *bla*_NDM-5_, *bla*_TEM-1B_, *dfrA12, erm(B), mdf(A), mph(A), rmtB, sul1, sul2* |
| **61464** |  | *aadA2, aph(3'')-Ib, aph(6)-Id*, *bla*_CTX-M-14_, *bla*_NDM-5_, *bla*_TEM-1B_, *dfrA12, erm(B), mdf(A), mph(A), rmtB, sul1, sul2* |
| **62757** |  | *aph(3'')-Ib, aph(6)-Id*, *bla*_CTX-M-14_, *bla*_NDM-5_, *bla*_TEM-1B_, *dfrA12, erm(B), mdf(A), mph(A), rmtB, sul1, sul2* |
| **62774** | IncHI2A, IncHI2, IncFII | *aac(3)-Iia, aac(6')-Ib-cr, aph(3'')-Ib, aph(6)-Id*, *bla*_CTX-M-14_, *bla*_CTX-M-15_, *bla*_NDM-5_, *bla*_OXA-1_, , *bla*_TEM-1B_,, *catA1, dfrA12, dfrA14, erm(B), mdf(A), mph(A), qnrB1, rmtB, sul1, sul2* |
| **64796** |  | *aadA2, aph(3'')-Ib, aph(6)-Id*, *bla*_CTX-M-14_, *bla*_NDM-5_, *bla*_TEM-1B_, *dfrA12, erm(B), mdf(A), mph(A), rmtB, sul1, sul2* |
| **GON1437** |  | *aadA2, aph(3'')-Ib, aph(6)-Id*, *bla*_CTX-M-14_, *bla*_NDM-5_,*dfrA12, mdf(A), sul1, sul2* |
| **JCH7040** |  | *aph(3'')-Ib, aph(6)-Id*, *bla*_CTX-M-14_, *bla*_NDM-5_, *bla*_TEM-1B_, *dfrA12, erm(B), mdf(A), mph(A), rmtB, sul1, sul2* |
| **JCH767** |  | *aadA2, aph(3'')-Ib, aph(6)-Id*, *bla*_CTX-M-14_, *bla*_NDM-5_, *bla*_TEM-1B_, *dfrA12, erm(B), mdf(A), mph(A), rmtB, sul1, sul2* |
| **JCH8249** |  | *aadA2, aph(3'')-Ib, aph(6)-Id*, *bla*_CTX-M-14_, *bla*_NDM-5_, *bla*_TEM-1B_, *dfrA12, erm(B), mdf(A), mph(A), rmtB, sul1, sul2* |
| **MOC653** |  | *aadA2, aph(3'')-Ib, aph(6)-Id*, *bla*_CTX-M-14_, *bla*_NDM-5_, *bla*_TEM-1B_, *dfrA12, erm(B), mdf(A), mph(A), rmtB, sul1, sul2* |
| **MOC8113** | IncHI2A, IncHI2 | *aac(6')-Ib-cr, ant(3'')-Ia, aph(3'')-Ib, aph(6)-Id*, *bla*_CTX-M-14_, *bla*_CTX-M-15_, *bla*_NDM-5_, *bla*_OXA-1_, *bla*_TEM-1B_, *catA1, dfrA12, dfrA14, erm(B), mdf(A), mph(A), qnrB1,* *rmtB, sul1, sul2* |
| **MOC9136** |  | *aadA2, aph(3'')-Ib, aph(6)-Id*, *bla*_CTX-M-14_, *bla*_NDM-5_, *bla*_TEM-1B_, *dfrA12, erm(B), mdf(A), mph(A), rmtB, sul1, sul2* |
